# Peripheral lymphocyte count as a prognostic marker in cervical cancer patients treated with immune checkpoint inhibitors: a retrospective study

**DOI:** 10.1186/s12885-025-15173-x

**Published:** 2025-11-12

**Authors:** Mihoko Dofutsu, Masahiro Aichi, Toshiyuki Itai, Satoru Shinoda, Natsuko Kamiya, Tamaki Cho, Yuki Ogawara, Yumi Ishidera, Yuichi Imai, Etsuko Miyagi, Taichi Mizushima

**Affiliations:** 1 Department of Obstetrics and Gynecology, Graduate School of Medicine, Yokohama City University School of Medicine, 3-9, Fukuura, Kanazawa-ku, Yokohama, 236-0004 Japan; 2 Department of Biostatistics, Yokohama City University School of Medicine, Yokohama, Japan

**Keywords:** Peripheral lymphocyte count, Progression-free survival, Immune-checkpoint inhibitors, Cervical cancer

## Abstract

**Background:**

Pembrolizumab, an immune checkpoint inhibitor (ICI), has revolutionized the treatment of recurrent cervical cancer; however, its benefits are limited. This study investigated whether peripheral lymphocyte count (PLC) is a prognostic factor for ICI in patients with advanced or recurrent cervical cancer.

**Methods:**

This retrospective study included 47 patients diagnosed with advanced or recurrent cervical cancer, who were treated with pembrolizumab at our hospital between September 2022 and December 2024. The primary outcome was progression-free survival (PFS). The PLC was measured using a blood test conducted immediately before the first administration of pembrolizumab. The optimal PLC cut-off value was determined by using the first quartile point of the PLC distribution. Based on this cut-off value, the patients were allocated into two groups: a normal PLC group (PLC^high^) and low PLC group (PLC^low^). The impact of PLC on PFS was assessed using a Cox proportional hazards regression model using inverse probability of treatment weighting method based on the propensity score, as well as the log-rank test.

**Results:**

The median age of the participants was 55 years (interquartile range, 34–84 years). The most common histological type was squamous cell carcinoma (60% of cases). The cut-off value for PLC was set at 710/µL based on the first quartile point. Twelve patients had PLC^low^ (26%) and 35 had PLC^high^ (74%). Low PLC was significantly associated with shorter PFS (hazard ratio [HR], 2.91, 95% confidence interval [CI], 1.23–6.91, *p* = 0.013). In the sensitivity analysis, low PLC was also significantly associated with shorter PFS (HR, 4.10; 95% CI, 1.81–9.29, *p* < 0.001).

**Conclusions:**

PLC may have potential as a prognostic marker for immunochemotherapy in patients with advanced or recurrent cervical cancer. Pre-treatment assessment of PLC could be helpful in identifying patients at risk of poor outcomes and in supporting clinical decision-making.

**Supplementary Information:**

The online version contains supplementary material available at 10.1186/s12885-025-15173-x.

## Background

Cervical cancer is the fourth most common malignancy among women worldwide, with approximately 600,000 new cases reported in 2020 [1]. Standard treatment options include radical surgery, concurrent chemoradiotherapy, palliative chemotherapy, and radiotherapy. Although early-stage disease is often curable, the prognosis of patients with recurrent or metastatic lesions remains extremely poor. The 5-year overall survival rate in this population is 16.5%, highlighting the need for more effective therapeutic approaches [2].

The pivotal KEYNOTE-826 trial demonstrated the efficacy of pembrolizumab, an ICI, in combination with platinum-based chemotherapy with or without bevacizumab in patients with recurrent or advanced cervical cancer. In the intention-to-treat population, this combination reduces the risk of mortality by 33% and extends median progression-free survival (PFS) from 8.2 to 10.4 months [3]. This regimen was approved in Japan as a novel treatment for patients with cervical cancer [4]. However, the clinical benefit remains limited to a particular subset of patients, highlighting the need to identify reliable prognostic markers.

Recent studies have focused on hematological parameters as potential biomarkers for ICI-treated patients. In patients with cervical cancer, elevated neutrophil-to-lymphocyte ratio (NLR) and platelet-to-lymphocyte ratio (PLR) have been associated with shorter PFS [5]. Similarly, in gastric cancer, NLR, PLR, and lymphocyte-to-monocyte ratio (LMR) have been linked to both PFS and overall survival [6].

Among these, the peripheral lymphocyte count (PLC) is a readily available, low-cost marker. A low PLC has been associated with poor outcomes in ovarian, renal, bladder, and rectal cancers [7–10] and ICI-treated non-small cell lung as well as head and neck cancers [11, 12]. However, the significance of PLC in patients with cervical cancer treated with ICI remains unclear.

This study aimed to investigate whether baseline PLC is associated with survival outcomes in patients with recurrent or metastatic cervical cancer treated with the KEYNOTE-826 regimen.

## Methods

### Patients

This retrospective study was approved by the Ethics Committee of Yokohama City University Hospital (Approval No. F241200001). Informed consent was obtained using the opt-out method in accordance with ethical guidelines for retrospective observational studies. Eligible patients were diagnosed with advanced or recurrent cervical cancer at our institution between September 2022 and December 2024 and received pembrolizumab-based chemotherapy. All included patients had histopathologically confirmed diagnoses, classified as FIGO 2021 stage IVB or histologically confirmed recurrence, were aged ≥ 20 years at the time of diagnosis. Patients with serious comorbidities were excluded to minimize potential confounding factors.

Collected data included age at diagnosis, body mass index (BMI), performance status (PS), TNM classification, FIGO stage, histological subtype, baseline laboratory values before the first dose of pembrolizumab (PLC, hemoglobin levels, white blood cell count, and serum albumin [g/dL] levels), tumor marker levels (squamous cell carcinoma antigen [SCC]), and history of radiotherapy. All patients underwent CT imaging before treatment. Combined Positive Score (CPS) values were collected from biopsy samples at the initial diagnosis or recurrence [13]. Hypoalbuminemia, reflecting malnutrition, was defined as a serum albumin level < 3.5 g/dL, based on the CONUT score [14].

### Treatment for cervical cancer

The patients received pembrolizumab-based chemotherapy according to the KEYNOTE-826 regimen [3]. The treatment comprised paclitaxel and cisplatin plus pembrolizumab administered every 3 weeks. Carboplatin was used instead of cisplatin in patients with renal impairment. Bevacizumab (15 mg/kg every 3 weeks) was administered when deemed appropriate only to patients without contraindications and was withheld when clinical concerns were present (e.g., high perforation/fistula risk after pelvic chemoradiation or bladder/rectal invasion, history or active thromboembolism, uncontrolled hypertension, or significant proteinuria) according to the criteria used in the KEYNOTE-826 regimen [15]. Tumor response was assessed every three cycles or upon tumor marker elevation using CT imaging and evaluated per immune-related RECIST 1.1 [16]. Chemotherapy dose reduction or discontinuation was based on the Common Terminology Criteria for Adverse Events v5.0. Treatment initiation required an absolute neutrophil count ≥ 1,500/mm³ and a platelet count ≥ 100,000/mm³. For grade 3 hematologic toxicity, therapy was paused and resumed with dose reduction after recovery, and grade 4 toxicity resulted in discontinuation. Non-hematological toxicities were similarly managed. Grade 2 immune-related adverse events (irAEs) were managed with temporary interruption and corticosteroids whereas grade ≥ 3 events led to treatment discontinuation.

### Statistical analyses

Patients were allocated into two groups based on peripheral lymphocyte count: PLC^high^ and PLC^low^ using the first quartile as the cutoff. Differences in clinicopathological characteristics between the groups were evaluated using the Mann-Whitney U test or chi-squared tests. The impact of PLC on PFS was assessed using Cox proportional hazards regression models with inverse probability of treatment weighting (IPTW) and the log-rank test. Covariates potentially affecting prognosis, including age and serum albumin levels, were adjusted using causal inference methods. Sensitivity analyses were adjusted for performance status and history of radiotherapy. In addition, to account for the potential influence of unmeasured confounders, we performed further sensitivity analyses including disease status (recurrent or metastatic), performance status, and history of radiotherapy. As a sensitivity analysis regarding cutoff values, we performed analyses using multiple thresholds, including the definition of lymphopenia at PLC < 1000/µL [17]. PFS was estimated using the Kaplan–Meier method, and differences between groups were assessed with the log-rank test. To assess robustness against unmeasured confounders, E-values were calculated from the IPTW-adjusted hazard ratios [18].

As an exploratory analysis, the impact of radiotherapy timing on PLC was evaluated by comparing three groups: no radiotherapy, radiotherapy > 1 year before pembrolizumab initiation, and radiotherapy < 1 year before pembrolizumab initiation. Pairwise comparisons of PLC were conducted between the no-radiotherapy group and each of the two radiotherapy groups (< 1 year and ≥ 1 year) using the Mann–Whitney U test. To adjust for multiple comparisons, Bonferroni correction was applied, and adjusted p-values were reported. The incidence of irAEs was compared between the PLC^high^ and PLC^low^ groups using the chi-square test. Covariate balance and additional visualizations are provided in Supplementary Figs. 1 and 2.

Statistical significance was set at *p* < 0.05. After Bonferroni correction, the significance threshold was set at *p* < 0.025. Statistical analyses were performed using JMP Pro version 15.0 (SAS Institute Inc., Cary, NC, USA).

### Endpoints

The primary endpoint was PFS, defined as the time from diagnosis to the date of radiologically confirmed disease progression or the most recent follow-up.

## Results

### Patient characteristics

Forty-seven patients with advanced or recurrent cervical cancer treated with the KEYNOTE-826 regimen between September 2022 and December 2024 were included in this retrospective study. All patients were pathologically diagnosed at our institution and either met the FIGO 2021 stage IVB criteria or had confirmed histological evidence of recurrence. The follow-up period ranged from 1 to 22 months, and no patients were lost to follow-up within 1 year of treatment initiation.

Table 1 presents the baseline patient characteristics. The median age of the patients was 56.8 years (SD, ± 12.3). The most common histological subtype was the squamous cell carcinoma (60%). The chemotherapy regimens included paclitaxel–cisplatin plus pembrolizumab in 21 patients (45%) and paclitaxel–carboplatin plus pembrolizumab in 26 patients (55%). Bevacizumab was administered to 33 (70%) patients. PFS data were collected at the time of data locking for the primary endpoint analysis. Table 1 summarizes baseline characteristics, including age, FIGO stage, histological subtype, TNM classification, CPS, prior treatment, and history of radiotherapy. Twenty-six patients (55%) had a history of radiotherapy, while 21 (45%) did not (Table 1).

**Table 1:** Patients and tumor characteristics

Baseline characteristics	All patients (*n* = 47)	PLC^low^ group (*n*=12)	PLC^high^ group (*n*=35)
Age at diagnosis, y, mean (SD)	56.8 (± 12.3)	56.9 (± 9.4)	56.7 (± 13.2)
BMI, kg/m^2^, Mean (SD)	21.6 (± 4.0)	19.2 (± 4.3)	22.3 (± 3.6)
	Numbers, (%)		
FIGO 2018 stage at initial diagnosis, n (%)			
I/II	13 (28)	3 (25)	10 (28)
III/IV	34 (72)	9 (75)	25 (71)
Histology, n (%)			
SCC	32 (68)	8 (67)	23 (66)
AD	10 (21)	3 (25)	7 (20)
Others	8 (17)	1 (8)	7 (20)
PD-L1 combined positive score, n (%)			
10 ≥	34 (72)	8 (67)	26 (74)
1–10	10 (21)	3 (25)	7 (20)
< 1	1 (2)	0 (0)	1 (3)
Unmeasurable	2 (4)	1 (8)	1 (3)
Malnutrition			
Yes (serum albumin < 3.5 g/dL)	14 (30)	3 (25)	11 (31)
No (serum albumin ≥ 3.5 g/dL)	33 (70)	9 (75)	24 (69)
ECOG performance status score, n (%)			
0	38 (80)	10 (84)	28 (80)
1	9 (20)	2 (16)	7 (20)
Disease status, n (%)			
Metastatic	14 (30)	0	14 (40)
Recurrent or persistent	33 (70)	12 (100)	21 (60)
History of radiotherapy, n (%)			
Yes	26 (55)	11 (92)	15 (43)
No	21 (45)	1 (8)	20 (57)
Treatment chemotherapy, n (%)			
Cisplatin-based	21 (45)	2 (16)	19 (54)
Carboplatin-based	26 (55)	10 (84)	16 (46)
Bevacizumab administration			
Yes	33 (70)	6 (50)	27 (77)
No	14 (30)	6 (50)	8 (23)
irAE			
Any grade	23 (49)	3 (25)	20 (57)

*Abbreviations*: *SD*, standard deviation; *PLC*, peripheral lymphocyte count; *BMI*, body mass index; *FIGO*, International Federation of Gynecology and Obstetrics; *SCC*, Squamous Cell Carcinoma; *AD*, Adenocarcinoma; *PD-L1*, Programmed death-ligand 1; ECOG Eastern Cooperative Oncology Group，*irAE*

### Baseline characteristics of patients grouped according to PLC

The cohort was further stratified based on PLC, using 710/µL as the optimal cut-off value, determined by the first quartile. Twelve patients (26%) were classified as PLC^low^, and 35 (74%) as PLC^high^. The baseline characteristics of the PLC group are detailed in Table 1.These findings are illustrated in Supplementary Figs. 1 and 2. All patients in the PLC^low^ group received immunotherapy for recurrent disease, whereas 34% of those in the PLC^high^ group received pembrolizumab as first-line therapy. A higher proportion of patients in the PLC^low^ group had a history of radiotherapy than those in the PLC^high^ group. Differences were also observed in the chemotherapy regimens; carboplatin-based regimens were more common in the PLC^low^ group, whereas cisplatin-based treatment was more frequent in the PLC^high^ group. Bevacizumab was administered to both groups, with slightly fewer patients in the PLC^low^ group.

### Prognostic impact of PLC on PFS

**Fig. 1:**
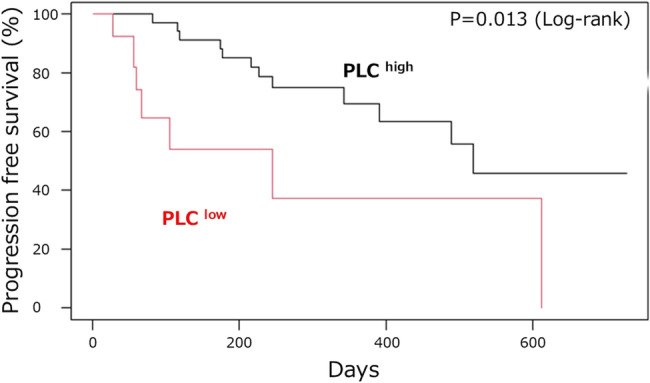
Kaplan–Meier Curves for Progression-Free Survival Adjusted for Age and Serum Albumin

**Fig. 2:**
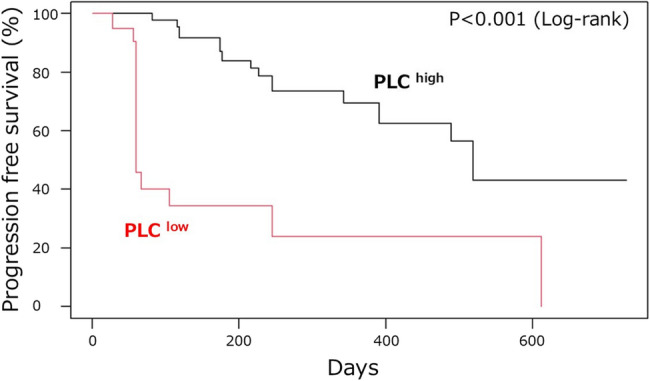
Sensitivity Analysis: Progression-Free Survival Adjusted for Age, Serum Albumin Levels, Performance Status, and Radiotherapy History

The median PFS was significantly shorter in the PLC^low^ group than that in the PLC^high^ group (245 vs. 519 days, *p* = 0.0284). After adjusting for age and nutritional status, low PLC remained significantly associated with shorter PFS (hazard ratio [HR] = 2.91, 95% confidence interval [CI]: 1.23–6.91, *p* = 0.013) (Fig. 1). Sensitivity analysis also confirmed that PLC low status was significantly associated with worse PFS (HR = 4.10, 95% CI: 1.81–9.29, *p* < 0.001) (Fig. 2). Even after adjustment for potential unmeasured confounders, low PLC status remained associated with a trend toward worse PFS (HR = 3.45, 95% CI: 1.42–8.36, *p* = 0.06). As sensitivity analyses regarding cutoff values, alternative PLC cutoff values were examined. At 600/µL, the trend remained but did not reach statistical significance, likely due to the small sample size (log-rank *p* = 0.155) (Supplementary Fig. 3 A). At 700/µL, PLC^low^ patients showed significantly shorter PFS compared with PLC^high^ (log-rank *p* = 0.048) (Supplementary Fig. 3B). By contrast, with the conventional definition of lymphopenia at 1,000/µL, no significant difference in PFS was observed (log-rank *p* = 0.61) (Supplementary Fig. 3 C).

### Relationships between radiotherapy and lymphopenia

**Fig. 3:**
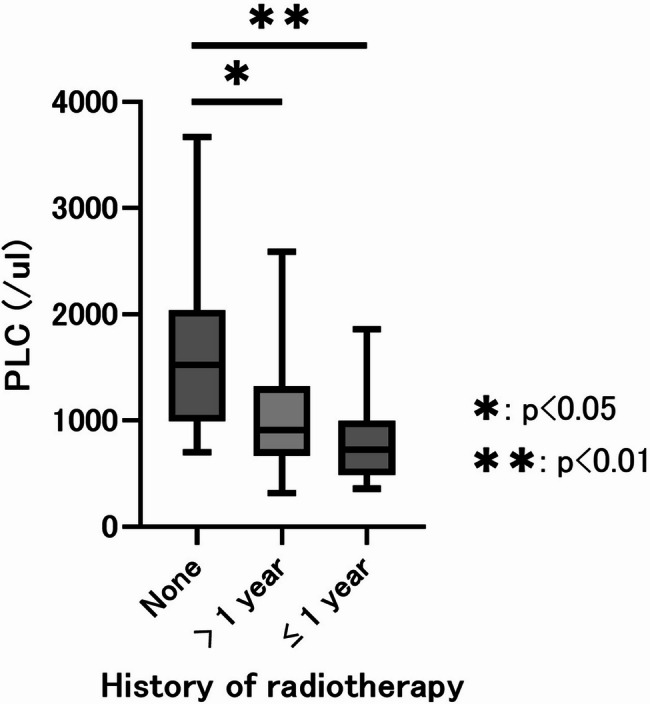
Box Plot of Pre-Treatment Lymphocyte Counts Based on Radiotherapy History Before ICI TreatmentPatients were categorized based on the timing of prior radiotherapy: no history of radiotherapy, radiotherapy >1 year before ICI treatment, and radiotherapy <1 year before ICI treatment

PLC values were compared among three subgroups: patients without a history of radiotherapy, those who received ICI ≥ 1 year after radiotherapy, and those who received ICI < 1 year after radiotherapy (Fig. 3). The median PLC was 1526/µL in patients without prior radiotherapy, 911/µL in those with ICI ≥ 1 year after radiotherapy, and 724/µL in those with ICI < 1 year after radiotherapy. Compared to the non-radiotherapy group, both the ≥ 1-year group (Bonferroni-adjusted *p* = 0.036) and < 1-year group (Bonferroni-adjusted *p* = 0.0016) had significantly lower PLC values.

### PLC and irAEs

The incidence of irAEs was significantly higher (*p* = 0.038) in the PLC^high^ group (57%) than in the PLC^low^ group (25%) (Table 2). Most events in the PLC^high^ group were grade 1–2, including dermatitis (20%), thyroid dysfunction (11%), and adrenal insufficiency (11%). Adrenal insufficiency (6%) and myositis (6%) were observed in the PLC^high^ group. This information is presented in detail in Table 2.

**Table 2:** Baseline characteristics of irAE in the PLC^Low^ and PLC^High^ groups

Baseline characteristics	PLC^low^ group (*n*=12)	PLC^high^ group (*n*=35)	*P* value
	Numbers, (%)		
irAE Any grade	3 (25)	20 (57)	0.038
Grade 1–2	2 (17)	16 (46)	
Grade ≥ 3	1 (8)	4 (11)	
Thyroid-related irAE Grade 1–2	0	4 (11)	
Thyroid-related irAE Grade ≥ 3	0	0	
Adrenal insufficiency Grade 1–2	0	4 (11)	
Adrenal insufficiency Grade ≥ 3	0	2 (6)	
Dermatitis Grade 1–2	0	7 (20)	
Dermatitis Grade ≥ 3	0	0	
Myositis Grade 1–2	1 (8)	1 (3)	
Myositis Grade ≥ 3	0	2 (6)	
Colitis Grade 1–2	1 (8)	0	
Colitis Grade ≥ 3	1 (8)	0	
Interstitial pneumonia Grade 1–2	0	0	
Interstitial pneumonia Grade ≥ 3	0	0	
Other irAE Grade 1–2	0	3 (7)	
Other irAE Grade ≥ 3	0	0	

*Abbreviations*: irAE: immune-related adverse events; Percentages in parentheses

## Discussion

In a retrospective cohort of patients with recurrent or advanced cervical cancer treated with pembrolizumab-based chemotherapy, lower pre-treatment PLC was significantly associated with a poorer prognosis. After adjusting for performance status and serum albumin levels, low pre-treatment PLC remained significantly associated with shorter PFS. This relationship was most pronounced in patients who underwent prior radiotherapy, suggesting that radiation-induced lymphopenia may compromise the efficacy of ICIs. Prognosis in recurrent cervical cancer remains poor despite radical surgery [19, 20], underscoring the need for reliable biomarkers. The PLC, routinely measured in patients receiving cervical cancer care, is a practical and accessible biomarker for predicting ICI efficacy.

### PLC and prognosis

In this study, patients with cervical cancer with pre-ICI PLC < 710 µL experienced significantly shorter PFS. These findings align with evidence from non–small cell lung cancer as well as head and neck squamous cell carcinoma showing that patients with low lymphocyte counts before starting ICI therapy have shorter PFS [11, 12]. Pre-treatment lymphopenia may indicate systemic immunosuppression, which can reduce the number of tumor-infiltrating lymphocytes and promote an immunosuppressive tumor microenvironment, thereby weakening the effectiveness of ICI [11, 21]. However, the mechanism through which lymphopenia causes resistance to ICI therapy remains unclear.

The lymphocyte count has been identified as a prognostic factor for various cancer treatments. Pre-treatment lymphopenia is associated with inferior outcomes in patients with cervical cancer undergoing concurrent chemoradiation therapy and in those with recurrent disease [21, 22]. These observations underscore lymphocyte count as a critical biomarker of antitumor immune competence in cancer therapy.

However, defining the optimal lymphocyte count cutoff remains a challenge. Although a PLC < 1,000/µL is widely used to define lymphopenia [17], this threshold has shown inconsistent results in ICI-treated patients: it has not been identified as a prognostic factor in melanoma cohorts [23], whereas in lung cancer cohorts it has been reported to be prognostic [24], and in our cohort, a PLC cutoff of 1,000/µL did not demonstrate prognostic value. It indicates the need for disease- and therapy-specific criteria. Future research should establish tailored cutoffs to improve risk stratification and guide personalized immunotherapy.

### PLC and radiotherapy

Low PLC were more common in patients with a history of radiotherapy. This finding is consistent with reports that radiation-induced lymphopenia adversely affects overall and PFS in patients with cervical cancer [25, 26]. Therefore, clinicians should consider radiation-induced lymphopenia when initiating ICI therapy in previously irradiated patients. One proposed mechanism involves the expansion of myeloid-derived suppressor cells following irradiation [27]. In a mouse model of radiation-induced lymphopenia, interleukin-7 administration restored CD8-positive tumor-infiltrating lymphocytes [28]. These data suggest that interventions targeting the tumor immune microenvironment may improve the outcomes in patients with radiation-induced lymphopenia.

In our cohort, patients who received ICIs within one year after radiotherapy had the lowest PLC, and a shorter interval between radiotherapy and ICI initiation appeared to exacerbate lymphopenia and potentially impair subsequent ICI efficacy. These findings are consistent with recent preclinical and clinical evidence indicating that persistent radiation-induced lymphopenia may adversely affect immunotherapy outcomes [25, 26]. This underscores the importance of considering the interval between radiotherapy and ICI initiation when treating patients with cervical cancer.

### PLC and irAE

Although this study did not prospectively evaluate PLC as a predictor of irAEs, patients with high PLC experienced significantly more irAEs than those with low PLC, consistent with prior reports linking lymphocyte levels to irAE risk [29–31]. ICIs expand T-cell repertoires and suppress regulatory T-cells, thereby increasing the number of autoreactive lymphocytes that trigger irAEs via cytokine release and autoantibody production, making elevated PLC a potential risk marker [31]. In our cohort, most irAEs in the PLChigh group were low-grade events such as dermatitis and thyroid dysfunction, whereas severe grade 3–4 events were rare. These findings suggest that elevated PLC may reflect heightened immune activation, predisposing patients to both improved antitumor efficacy and increased irAE risk. However, the limited sample size precludes definitive conclusions regarding specific irAE subtypes. Clinically, if validated, baseline PLC may inform treatment selection and supportive care. Patients with lymphopenia might merit lymphocyte-sparing strategies—such as avoiding unnecessary corticosteroids or using radiotherapy plans that minimize lymphocyte dose—and could benefit from adjunctive measures including nutritional support or exploratory IL-7–based interventions [32, 33].

### Strengths of the study and future prospects

The strengths of this study include its novel demonstration that pre-treatment PLC is significantly associated with prognosis in patients with cervical cancer receiving ICI therapy, highlighting its potential utility for risk stratification and treatment planning in future prospective trials. Second, identifying this biomarker may inform evolving treatment paradigms, particularly as combined radiotherapy and ICI regimens gain traction following the success of the KEYNOTE-A18 trial in guiding treatment decisions of patients with lymphopenia. Finally, our findings suggest that interventions targeting radiation-induced lymphopenia could improve outcomes, and elucidating the underlying mechanisms may reveal actionable targets for pharmacological modulation.

### Limitations

First, the retrospective design was susceptible to selection bias. Second, the relatively small sample size explicitly limited statistical power and reduced the generalizability of the results; notably, all patients in the PLC^low^ group had recurrent disease, preventing us from distinguishing whether lymphopenia was a feature of tumor recurrence or a chance finding. The KEYNOTE-048 trial in recurrent head and neck squamous cell carcinoma reported a hazard ratio of 1.05 for overall survival with pembrolizumab in recurrent case [34], suggesting limited efficacy in this setting. Third, our single-center Asian cohort may have limited the external validity of the findings. Finally, the follow-up duration (22 months) may be insufficient for robust assessment of long-term outcomes. Given these limitations, our findings need to be validated in large-scale prospective studies.

## Conclusions

This study is the first to demonstrate a significant association between pre-treatment lymphocyte count and prognosis in a cohort of patients with recurrent or advanced cervical cancer who received pembrolizumab-based chemotherapy. Future multicenter studies should expand the sample size and compare multiple cut-off values to identify optimal thresholds. Additionally, the efficacy of prophylactic immunostimulatory interventions in patients with lymphopenia should be evaluated, along with serial immune profiling, to elucidate the underlying mechanisms.

## Supplementary Information

Supplementary Material 1.

Supplementary Material 2.

Supplementary Material 3.

## Data Availability

Raw data were generated at the Yokohama City University School of Medicine. Data supporting the findings of this study are available from the corresponding author, Masahiro Aichi, upon request.

## Abbreviations

BMI: Body mass index
CI: Confidence interval
CPS: Combined positive score
CT: Computed tomography
FIGO: International Federation of Gynecology and Obstetrics
HR: Hazard ratio
ICI: Immune checkpoint inhibitor
irAE: Immune-related adverse event
IPTW: Inverse probability of treatment weighting
LMR: Lymphocyte-to-monocyte ratio
NLR: Neutrophil-to-lymphocyte ratio
PFS: Progression-free survival
PLC: Peripheral lymphocyte count
PS: Performance status
SCC: Squamous cell carcinoma antigen
SD: Standard deviation
TNM: Tumor, Node, Metastasis classification
