# Supplementary figures and images for: Peripheral lymphocyte count as a prognostic marker in cervical cancer patients treated with immune checkpoint inhibitors: a retrospective study
Source: BMC Cancer. 2025 Nov 12;25:1762. doi: 10.1186/s12885-025-15173-x (PMC12613785; doi:10.1186/s12885-025-15173-x)

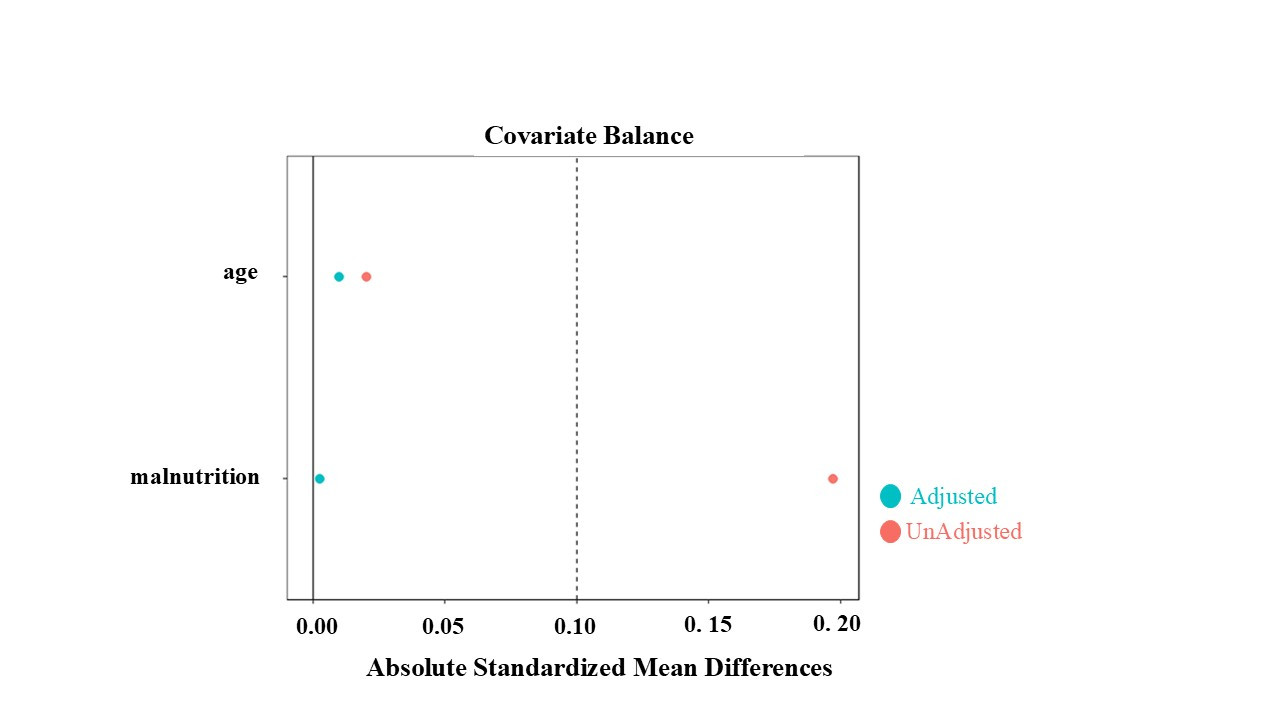

Supplement: Supplementary file 1 — Supplementary Material 1. [file 12885_2025_15173_MOESM1_ESM.jpg]

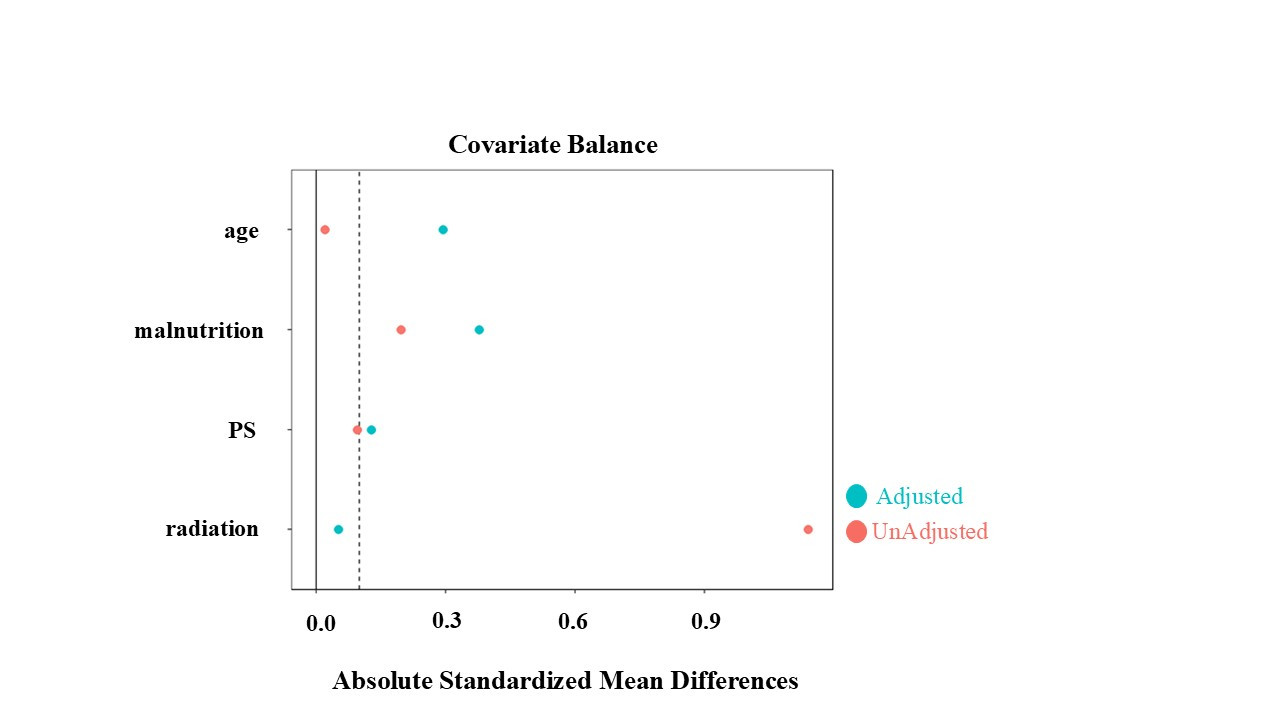

Supplement: Supplementary file 2 — Supplementary Material 2. [file 12885_2025_15173_MOESM2_ESM.jpg]

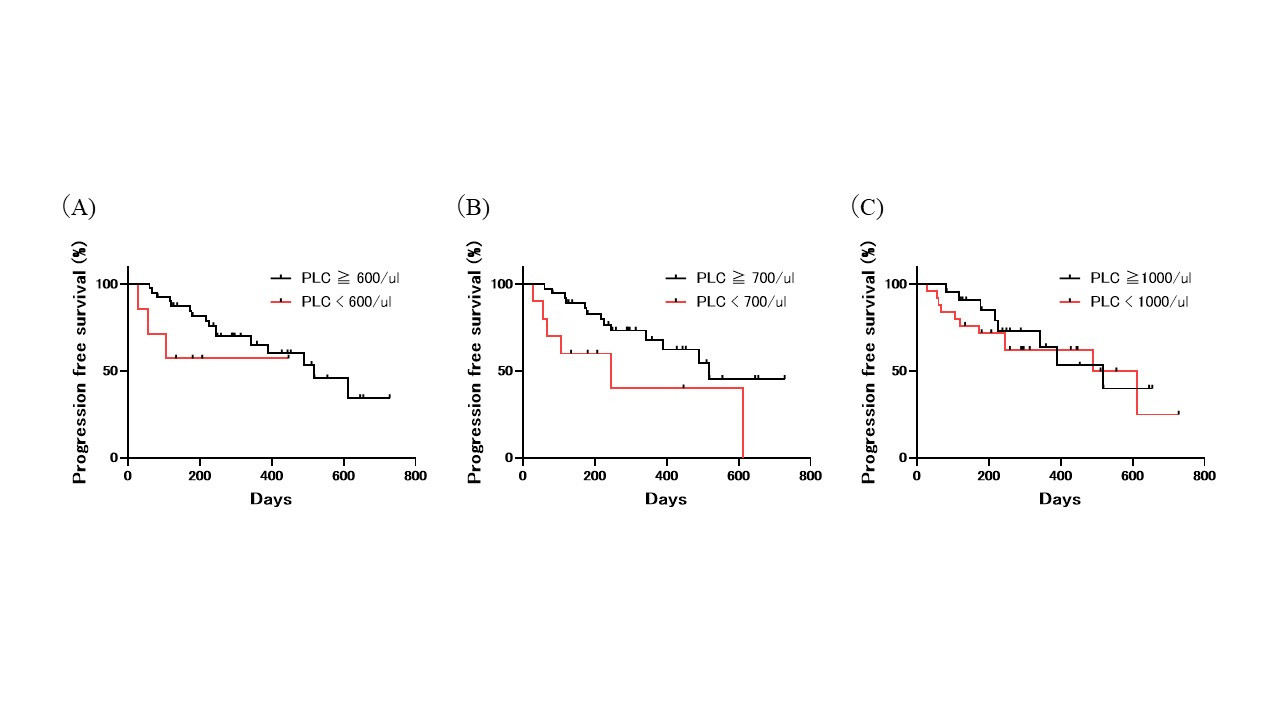

Supplement: Supplementary file 3 — Supplementary Material 3. [file 12885_2025_15173_MOESM3_ESM.jpg]
